# Associations with the inter-recti distance at gestation week 37: a prospective cohort study among healthy pregnant women

**DOI:** 10.1186/s12884-025-07741-7

**Published:** 2025-05-29

**Authors:** Nina-Margrethe Theodorsen, Rolf Moe-Nilssen, Kari Bø, Inger Haukenes

**Affiliations:** 1 Department of Global Public Health and Primary Care, University of Bergen, PO. Box 7804, Bergen, NO-5020 Norway; 2 Department of Sports Medicine, Norwegian School of Sport Sciences, PO. Box 4014, Ullevål stadion, Oslo, 0806 Norway; 3 Department of Obstetrics and Gynecology, Akershus University Hospital, Lørenskog, Norway; 4 Research Unit for General Practice, Norce – Norwegian Research Centre, Bergen, Norway

**Keywords:** Pregnancy, Inter-recti distance, Diastasis recti abdominis, Ultrasonography

## Abstract

**Background:**

During pregnancy the inter-recti distance (IRD) increases. There is, however, limited knowledge of what factors may contribute to this increase.

**Objective:**

To investigate associations between sociodemographic characteristics, co-existing health conditions and pregnancy related factors in pregnant women before gestation week 20, and IRD at gestation week 37.

**Methods:**

One hundred and twenty pregnant women provided baseline information on sociodemographic characteristics, co-existing health conditions, and pregnancy-related factors. Baseline IRD (gestation week 16–19) and IRD at gestation week 37 (outcome) were measured with ultrasound 2 cm above the umbilicus. Associations between baseline variables and IRD at gestation week 37 were investigated with bivariable regression analyses, and only variables statistically significantly associated with outcome were included in a multivariable linear regression analysis. The level of significance was set at 0.05.

**Results:**

Bivariable associations between baseline variables and IRD at gestation week 37 were statistically significant for BMI, waist circumference, parity, stretch marks (striae), varicose veins and IRD. In the final multivariable linear regression analysis only baseline IRD was significantly associated with IRD at gestation week 37 (B = 0.8, SE = 0.1, 95% CI = 0.6–1.0, *P*-value < 0.001, R^2^ = 0.384).

**Conclusion:**

No sociodemographic characteristics, co-existing health conditions or other significant related factors present before gestation week 20 other than baseline IRD was significantly associated with IRD at gestation week 37, with the clinical implication that for each 1 mm increase in baseline IRD, there was an average increase of 0.8 mm in IRD at gestation week 37. Approximately 38% of the variance in IRD at gestation week 37 is explained by baseline IRD and other included variables.

**Supplementary Information:**

The online version contains supplementary material available at 10.1186/s12884-025-07741-7.

## Background

During pregnancy a woman’s body undergoes several changes, with the increased size of the abdomen as the most profound morphological alteration [1, 2]. This is a completely normal and necessary development caused by the increased weight and size of the uterus accommodating to the growing fetus. The alteration causes not only the abdominal muscles to stretch, but also the linea alba. The linea alba is a dense connective tissue structure that spans the space between the two bellies of the rectus abdominis muscle. The inter-recti distance (IRD) refers to the width of this space, measured between the medial borders of the rectus abdominis muscle. During the second trimester (gestation week 14–27) the uterus grows and is approximately at the level of the umbilicus towards gestation week 20. It is assumed that the IRD increases from this stage and throughout the third trimester (gestation week 28–42) mostly due to the growing uterus [1–3]. This increase in IRD naturally resolves for most women postpartum, however it is considered normal that the IRD remains somewhat increased compared to pre-pregnancy state [3–5]. For approximately 30% of women 12 months postpartum the IRD has not returned to these expected normal values [6].

When the linea alba is stretched beyond its original size, it gives name to a common condition in pregnant and postpartum women; diastasis recti abdominis (DRA). Prevalence of DRA during the last trimester of pregnancy has been reported to be from 60 to 100% [4–6]. There is however no consensus for when an IRD is considered a clinically significant DRA. Previous studies consider an IRD to be pathological when the separation is more than 2 cm [3, 7, 8], whilst a more recent study suggests this ought to be increased to 3.4 cm [9]. There is limited knowledge of the increase in IRD during pregnancy, and what are considered normal values towards end of pregnancy. In addition, studies vary in quality, measurement locations, measurement modality and procedures differ, which make comparisons difficult [1–6]. Two-dimensional ultrasonography is recommended for measuring and monitoring change in IRD over time, as it is reported to be both reliable and valid [10]. The IRD is reported to be largest 2 cm above the umbilicus and decreases towards the xiphoid process and below the umbilicus [4]. Short term experimental studies have shown that headlift and abdominal crunch decreases the IRD significantly compared to resting position during one single contraction, in both pregnant and postpartum women which implies that the measurement position will affect the measurement outcome [11–13]. To our knowledge no studies have measured the IRD in the same individuals at different timepoints during pregnancy, and most studies have been done on a postpartum population, without investigating potential factors associated with increased IRD throughout pregnancy.

Factors such as age, weight, height, waist circumference, heavy lifting, parity, IRD, hypermobility, comorbidity, stretch marks, varicose veins abdominal and pelvic floor muscle exercise, presence of urinary incontinence, pelvic girdle pain and low back pain have been suggested in the literature to be associated with increased IRD postpartum [1, 4, 6, 9, 14]. There is however conflicting evidence for these associations. Recent studies found no associations between presence of urinary incontinence, pelvic girdle pain and low back pain and increased IRD [4, 6, 15, 16]. One study suggests that parity, body mass index (BMI) and diabetes are the most plausible risk factors for increased IRD [14] and Boissonnault & Blaschak (1987) concludes with a strong association between advancing pregnancy and increased IRD postpartum, and that weak abdominal muscles is a contributing factor [1]. This is in line with findings from Gluppe et al. (2021) concluding that postpartum women with DRA might have weaker abdominal muscles than postpartum women without DRA [15]. Kaufmann et al. (2021) presents increased age, increased BMI and parity as independent risk factors for increased IRD in adults [9].

However, we do not know to what extent these factors are associated with IRD late in pregnancy. As the IRD usually increases during the last two trimesters of pregnancy [1, 4, 6] it is important to conduct studies investigating which factors may contribute to increased IRD at 37 weeks of gestation. This will add to our knowledge of DRA and whether it can be prevented at an early stage.

## Aim

The aim of this study was to investigate associations between sociodemographic, co-existing health conditions and pregnancy related factors in women during the early stages of the second trimester, and IRD at gestation week 37.

## Methods

### Design

The present study has a prospective cohort design, where baseline variables were collected prior to gestation week 20 (gestation week 16–19) and outcome at gestation week 37. Procedures agreed with the Helsinki Declaration, and ethical approval was given by the Norwegian Regional Ethics Committee (76296 2020/2304). All participants received oral and written information about the study and signed a consent form prior to inclusion. The study is reported in adherence to STROBE [17].

### Participants

Pregnant women were recruited from local health centers, physiotherapy clinics and the social media platforms Facebook and Instagram. Inclusion criteria were pregnancy prior to gestation week 20, and age 18 years and older. Exclusion criteria were inability to understand Scandinavian languages, pregnancy related serious illnesses regarding both fetus and/or mother, chronic physical or mental illness, and stillbirth or premature births prior to gestation week 37. One hundred and thirty-seven pregnant women were enrolled in the study. Two participants gave birth prior to gestation week 37 and were therefore excluded. Three participants withdrew from the study without reason, and twelve participants were unable to attend an assessment during gestation week 37 due to covid restrictions and were also excluded. One hundred and twenty pregnant women completed both assessments and were included in the study.

### Outcome measure

The outcome measure was IRD (mm) at gestation week 37, measured using a two-dimensional ultrasonography with a diagnostic scanner with a linear probe (Alpinion EC8Diamond, L8-17 MHz transducer). Images were taken 2 cm above the umbilicus with the upper edge of the umbilicus as point of reference [18]. The location was marked on the skin with pen prior to imaging. Images where the IRD was too large to be captured using standardized brightness mode (B-mode), panoramic function was applied. All images were taken at end of exhalation and in accordance with procedures described by Teyhen et al. (2005) [19]. Participants rested supine on a plinth, with a pillow underneath the head, arms alongside the body and knees on a half-moon pillow cushion. The images were stored as Digital Imaging and Communications in Medicine (DICOM) format on a SAFE server. Measurements were performed using the free MATLAB Image Processing Toolbox programme. The IRD was defined as the shortest transverse lineal distance between the two medial borders of the rectus abdominis muscle (outside the hyperechoic line). The assessor was blinded to the participants when measuring the IRD, as participants’ identification had been replaced by a number prior to the measurement process. One assessor performed all assessments and measurements, a woman’s health physiotherapist with extensive clinical experience, specific training in the use of ultrasound and research experience of ultrasound imaging of the IRD in pregnant and postpartum women.

### Exposure variables

Exposure variables were collected prior to gestation week 20, during gestation week 16 and 19. All participants completed a self-administrated questionnaire with questions related to sociodemographic, co-existing health conditions and pregnancy-related factors (Supplementary file 1).

### Sociodemographic variables

Age (*years*), height (*cm*), weight (*kg*) was self-reported, and BMI estimated (*kg/m*^*2*^). Level of education was reported in four categories and were re-coded into two categories *low* (primary, secondary and college levels), and “high” (all levels of university degrees). Levels of regular physical activity for the last 4 weeks were reported as never, less than once a week, once per week, 2–3 times a week and almost every day and re-coded to *less than 3 times per week* and *≥ 3 times per week*. Abdominal exercise last 4 weeks were reported in 5 categories (never, 1–3 times, once a week, twice a week, 3 times or more per week) and re-coded *to less than 2 times per week* and *≥ 2 times per week*, and pelvic floor exercise was reported in 5 categories (never, once per week, twice per week, three times pr week or every day), and were re-coded *to less than 2 times per week* and *≥ 2 times per week*. Self-reported Quality of health was reported by using a simple, but valid epidemiologic variable by asking “how is your health now?” [20, 21] There were four alternative answers (poor, not good, good and very good) and re-coded into two categories, *poor* (poor and not good) or *good* (good and very good).

### Co-existing health condition variables

Presence of urinary incontinence and pelvic girdle pain were assessed using the International Consultation on Urinary Incontinence Short form questionnaire (ICIQ_UI-SF) [22], and the Pelvic Girdle Questionnaire (PGQ) [23], respectively. The ICIQ_UI-SF is reported to have moderate convergent validity, good discriminant validity, and good reliability and the PGQ has reportedly both high reliability and validity and is a condition specific questionnaire for both pre- and postpartum women [22, 23]. Presence of urinary incontinence and pelvic girdle pain was re-coded to either *yes or no*, were “no” was a score zero and “yes” was any score above zero. Hypermobility was reported using the five-part questionnaire on hypermobility (5PQ) [24], and as the main aim was to determine whether generalized joint hypermobility in early pregnancy was present or not, the 5PQ was considered valid [25]. The 5PQ was re-coded to *yes if two yes-answers* or *more*, else *no* [24]. Stretchmarks, varicose veins and rheumatological disease were reported as *yes* or *no*. Presence of low back pain was reported using the reliable and valid Modified Oswestry Disability Index [26] which scores levels of self-reported disability associated with presence of low back pain. *The scores range from 0 to 100%.* This questionnaire has not been tested specifically for a pregnant population.

### Pregnancy-related variables

The IRD *(mm)* was measured as described in the ‘Outcome’ paragraph.

The symphysis fundal height (SFH) (*cm*) is the distance from top of the uterine fundus to the top of the symphysis pubis [27, 28]. This is an international standard measure for both fetal growth monitoring and estimation of gestational age [27, 29]. During the measurement the participant was supine lying on the plinth, arms resting alongside body, knees resting on a half-moon pillow cushion. The top of the uterine fundus was palpated, and the non-elastic tape measure was secured at the fundus with one hand, centimeters facing down to reduce bias. The tape measure was kept in contact with the skin and measured diagonally to the top of the symphysis pubis [28].

Waist circumference (*cm*) was measured with the participant in standing position. A tape measure was placed in a horizontal plane around the abdomen mid-way between the lower rib and iliac crest, just above the umbilicus. The tape measure was held snug to the skin, but did not compress the skin [30].

Gestation week was self-reported (*weeks 16–19*). Parity was reported as numbers of births and re-coded to *primi- and multigravida*. Number of fetuses were reported as numbers and re-coded to *single and multiple*. Delivery mode was reported and coded as previous *vaginal or c-section*.

### Statistical analysis

SPSS (version 29; IBM corporation) was used for all the statistical analyses.

Baseline variables were analyzed and presented as means with standard deviations (SD) or as numbers (N) with percentages (%). Associations between baseline variables and IRD at gestation week 37 (the outcome) were initially examined using bivariable linear regression analyses. Baseline variables that did *not* show a statistically significant association with the outcome (*p* >.05) were excluded from further analysis. Consequently, *only* those baseline variables that were significantly associated with the outcome were included in subsequent analyses.

In the further analysis, we first adjusted for baseline IRD in the bivariable associations to better understand the impact of baseline IRD on factors potentially associated with IRD at gestation week 37. Following this, we employed a multivariable linear regression model.

Associations are reported as unstandardized betas (B) with standard errors (SE), confidence intervals (CI), *p*-values and coefficient of determination (R^2^). The level of significance was set at 0.05. Linear plots and residual plots confirmed linearity between independent variables and the dependent variable. We performed Kolmogorov-Smirnov test for normality. All data was normally distributed, except for the continuous variables baseline IRD, age, and waist circumference. However, the deviation from normality for these variables was moderate and could be tolerated. Variance Inflation Factor (VIF) values ranged between 1.0 and 1.4, suggesting that multicollinearity was not a concern.

## Results

The mean age was 32 years (SD 3.6) and average BMI 25 (SD 4.1). About half of the participants reported low on educational level (47%). With respect to physical activity the majority were active more than 3 times per week (77%), whereas a minority performed specific abdominal or pelvic floor exercises regularly (abdominal 10%, and pelvic floor 11%). Only one fourth (25%) had work involving heavy lifting (> 5 kg) (Table 1).

**Table 1:** Distribution of sociodemographic, co-existing health conditions and pregnancy related variables at baseline (gestation week < 20) and bivariable associations with IRD at gestation week 37 among the study population (*N* = 120)

Variables		Mean	SD	B	SE	95% CI	*p*-value	*R* ^2^
**Sociodemographic**								
	Age (years)	32.1	3.6	0.3	0.4	− 0.5–1.0	0.477	0.004
	Height (cm)	168	6.0	0.0	0.2	− 0.4 − 0.5	0.862	0.000
	Weight (kg)	71.2	13.2	0.2	0.1	− 0.002 − 0.390	0.052	0.032
	BMI	25.1	4.1	0.6	0.3	0.003–1.3*	0.045	0.034
		**N**	**%**	**B**	**SE**	**95% CI**	***p*** **-value**	**R** ^**2**^
	Education^1^							
	Lower (vs. higher)	56	(47)	-2.1	2.7	-7.3–3.2	0.434	0.005
	Weekly physical activity							
	< 3 times (vs. ≥ 3)	28	(23)	-4.4	3.1	-10.5–1.8	0.163	0.016
	Weekly Abdominal exercise							
	< 2 times (vs. ≥ 2)	90	(75)	-1.1	3.1	-7.1–5.0	0.732	0.001
	Weekly Pelvic floor exercise							
	< 3 times (vs. ≥ 3)	89	(74)	4.9	3.0	-1.0–10.8	0.103	0.022
	Heavy lifting > 5 kg at work							
	Yes (vs. no)	30	(25)	-3.6	3.0	-9.6–2.4	0.239	0.012
	Self-perceived quality of health							
	Poor (vs. good)	22	(18)	6.3	3.4	− 0.4–13.0	0.065	0.029
**Co-existing health conditions**		**N**	**%**	**B**	**SE**	**95% CI**	***p*** **-value**	**R** ^**2**^
	Urinary Incontinence							
	Yes (vs. no)	50	(42)	0.1	2.7	-5.3–5.4	0.983	0.000
	Pelvic girdle pain							
	Yes (vs. no)	85	(71)	4.7	2.9	-1.1–10.4	0.109	0.022
	Hypermobility							
	Yes (vs. no)	24	(20)	− 0.3	3.3	-6.2–6.9	0.917	0.000
	Stretchmarks							
	Yes (vs. no)	51	(43)	5.9	2.6	0.7–11.1	0.026	0.041
	Varicose veins							
	Yes (vs. no)	22	(18)	6.9	3.4	0.2–13.5	0.043	0.034
	Rheumatological disease							
	Yes (vs. no)	6	(5)	15.6	5.9	3.9–27.3	0.009	0.056
		**Mean**	**SD**	**B**	**SE**	**95% CI**	***p*** **-value**	**R** ^**2**^
	Low back pain							
	Modified Oswestry	8.6	9.4	0.1	0.1	0.6 - − 0.2	0.620	0.002
**Pregnancy related**								
	IRD (mm)	27.2	10.3	0.8	0.1	0.6–1.0	< 0.001	0.335
	SFH^2^ (*n* = 113) (cm)	18.2	3.6	− 0.5	0.4	-1.2–0.2	0.146	0.019
	Waist circumference (cm)	94.3	10.1	0.3	0.1	0.031–0.541*	0.028	0.040
	Gestation week	18.4	1.2	-1.2	1.1	-3.3–0.9	0.270	0.010
		**N**	**%**	**B**	**SE**	**95% CI**	***p*** **-value**	**R** ^**2**^
	Parity							
	Multigravida (vs. primi)	59	(49)	6.0	2.6	0.9–11.1	0.023	0.043
	Number of fetuses							
	Multiple (vs. single)	2	(2)					
	Delivery mode^3^							
	Previous section (vs. vaginal)	2	(4)					

IRD = inter recti distance, N = number, BMI = body mass index, SD = standard deviation,

B = unstandardized beta, SE = standard error, CI = confidence interval, SD = standard deviation,

R^2^ = R square, vs. = versus

^1^High education = university level

^2^*N* = 113, not possible to measure the SFH in 7 participants

^3^*N* = 57

*Include 3 digits to demonstrate significant association with the outcome

Mean IRD at baseline was 27 mm (SD 10), and mean IRD at gestation week 37 was 52 mm (SD 15). At baseline 61 (51%) of the 120 participants presented with an IRD ≥ 2.8 cm measured 2 cm above the umbilicus. At gestation week 37, 119 (99%) presented with an IRD ≥ 2.8 cm measured 2 cm above the umbilicus. It was not possible to palpate the top of the uterine fundus to measure the SFH in 7 participants. Approximately half of the participants (49%) had given birth previously and only two of these had had a c-section. Only two of the participants carried more than one fetus. Approximately 70% reported presence of pelvic girdle pain, and 42% reported presence of urinary incontinence. The mean score of disability caused by low back pain was 8% on the modified Oswestry disability index. Only 5 participants reported a presence of rheumatological disease.

Bivariable associations between baseline variables and the outcome (IRD at gestation week 37) were statistically significant for baseline IRD (B = 0.8, 95% CI: 0.6-1.0), parity (B = 6.0, 95% CI: 0.9–11.1), stretch marks (B = 5.9, 95% CI: 0.7–11.1), and varicose veins (B = 6.9, 95% CI: 0.2–13.5) (Table 1). BMI and waist circumference were also significantly associated with the outcome, as demonstrated when including three digits in the CI’s (B = 0.6, 95% CI: 0.003-1.3) (B = 0.3, 95% CI: 0.031-0.5, respectively). In addition, rheumatological disease was significantly associated with the outcome; however, due to low numbers reporting the disease (*n* = 5) this variable was not included in further analyses presented in Table 2. There were only two participants carrying multiple fetuses, and only two participants had had previous c-section, and hence these data were also not included in the analysis. Explained variance (R^2^) for IRD baseline was 0.335, and for the remaining variables R^2^ ranged from 0.034 to 0.043 (Table 2). Adjustments for baseline IRD attenuated the bivariable associations (BMI, waist circumference and parity) to insignificant levels, whereas stretch marks (B = 4.8, 95% CI: 0.5–9.1) and varicose veins (B = 5.9, 95% CI: 0.4–11.3) were still significantly associated with the outcome (Table 2).

**Table 2:** Bivariate and multivariate associations between baseline variables (gestation week < 20) and IRD at gestation week 37

	Bivariate associations						Bivariate associations adjusted for IRD baseline						Multivariate associations				
	B	SE	95% CI	*p*- value	*R* ^2^		B	SE	95% CI	*p*- value	*R* ^2^		B	SE	95% CI	*p*- value	*R* ^2^
**IRD (mm)**	0.8	0.1	0.6 − 1.0	< 0.001	0.335								0.8	0.1	0.6 − 1.0	< 0.001	0.384
**BMI**	0.6	0.3	0.0-1.3	0.045	0.034		0.4	0.3	− 0.2-0.9	0.175	0.346		0.3	0.4	0.5-1.2	0.443	
**Waist circumference (cm)**	0.3	0.1	0.0-0.6	0.028	0.040		0.1	0.1	− 0.1-0.3	0.306	0.341		− 0.4	0.2	− 0.4-0.3	0.814	
**Parity (births)**																	
Multigravida (vs. primi)	6.0	2.6	0.9-11.1	0.023	0.043		− 0.1	3.7	-7.6-7.4	0.978	0.335		− 0.4	2.3	-4.9-4.2	0.879	
**Stretch marks**																	
Yes (vs. no)	5.9	2.6	0.7-11.1	0.026	0.041		4.8	2.2	0.5-9.1	0.028	0.362		3.8	2.2	− 0.6-8.2	0.091	
**Varicose veins**																	
Yes (vs. no)	6.9	3.4	0.2-13.5	0.043	0.034		5.9	2.8	0.4-11.3	0.035	0.360		4.7	2.8	− 0.9-10.2	0.100	

BMI = body mass index, IRD = inter recti distance, B = unstandardized beta, SE = standard error, CI = confidence interval, R^2^ = R square, vs. = versus

Results from the multivariate linear regression analysis showed that the association between IRD baseline and IRD at gestation week 37 was statistically significant (B = 0.8, 95% CI: 0.6-1.0). This indicates that for each 1 mm increase in baseline IRD, there was an average increase of 0.8 mm in IRD at gestation week 37 (Table 2). Additionally, approximately 38% of the variance in IRD at gestation week 37 is explained by the baseline IRD and other included variables.

## Discussion

The results of this prospective cohort study with 120 participants revealed that IRD measured prior to gestation week 20 (baseline) had a significant linear relationship with IRD late in pregnancy (gestation week 37), whereas sociodemographic variables, co-existing health conditions, and other pregnancy related variables were not associated. Moreover, we found that 34% of the variance in the IRD at gestation week 37 was explained solely by IRD at baseline and increased with only 4–38% when including baseline BMI, waist circumference, parity, stretch marks and varicose veins in the multivariable regression analyses. This implies that about 60% of the variance in IRD late in pregnancy was not explained by the model.

To our knowledge this is the first study with a prospective design following a cohort of pregnant women from early stages of second trimester throughout the third trimester using ultrasonography to measure IRD at gestation week 37 and examine potential associated factors, including IRD early in pregnancy. Previous studies have investigated possible predictors for IRD postpartum, measuring the IRD only once during pregnancy [3, 5], thus failing to examine the potential relationship between early and late IRD in pregnancy. In the present study we found that these two IRD measures were highly associated, indicating that the IRD during pregnancy may be relatively unaffected by extraneous factors.

Prevalence of DRA (IRD > 2.8 cm) in this present study was 51% measured 2 cm above the umbilicus, and 23% below the umbilicus at baseline (< gestation week 20) and 99% 2 cm above the umbilicus and 98% 2 cm below the umbilicus at gestation week 37. This corresponds with the findings of both Sperstad et al. (2016) and Mota et al. (2015). Sperstad et al. (2015) found a prevalence of DRA in 33% of the pregnant women at gestation week 21. They measured the IRD with palpation with finger widths, with a cut-off of two finger widths [6]. However, this is not a reliable measurement method when monitoring change in IRD and is less discriminative than ultrasound and therefore not directly comparable to our study. Mota et al. (2015) found a prevalence of DRA in 100% of their study population at gestation week 35, but they used a lower cut-off point for IRD (> 1.62 cm) and only measured the IRD 2 cm below the umbilicus [3]. Guo et al. (2024) also found a prevalence of 100% towards end pregnancy, IRD was measured by ultrasound imaging between 4.5 cm above and below the umbilicus [5].

BMI, weight, height, parity, waist circumference, abdominal exercise, hypermobility, previous caesarean section, low back pain and heavy lifting are all factors either believed or found to correlate with IRD in previous studies investigating risk factors for DRA [1, 4, 6, 9, 14]. Mota et al. (2015) found no link to any of the investigated variables and concluded that DRA was not linked to lumbo-pelvic pain postpartum [4]. These findings were supported by Sperstad et al. (2016) and Kaufmann et al. (2021). Kaufmann et al. (2021) found a correlation between high age (*p* =.001), increased BMI (*p* <.001) and parity (*p* <.037) and presence of DRA in adults [9]. Sperstad et al. 2016) found heavy lifting to correlate with presence of DRA 12 months postpartum. They do however interpret this finding with caution as the data was self-reported, not defined or measured directly [6]. Sperstad et al. found no link between lumbo-pelvic pain and presence of DRA [6]. Both Cavalli et al. (2021) and Kaufmann et al. (2021) conclude that pregnancy itself and increased BMI are the main risk factors for DRA [9, 14].

In the present study we found that BMI, waist circumference, parity, stretch marks and varicose veins early in the pregnancy were significantly associated with IRD at gestation week 37 in bivariable associations, but failed to reach a significant level in the multivariable model where IRD at baseline also was included. Interestingly height, abdominal exercise, hypermobility, previous caesarean section, heavy lifting and back pain were not associated with IRD at gestation week 37. Both Mota et al. (2015) and Sperstad et al. (2016) reported no link between DRA and lumbopelvic pain. In both studies low back pain and/or pelvic girdle pain were merged into one term [4, 6]. This makes it again difficult to compare results. It is however important to take into consideration the differences between these studies as this present study has investigated possible associated factors for increased IRD during pregnancy, while the other studies have investigated predictors for DRA postpartum.

Strengths of this exploratory study were the longitudinal design with IRD measurements at two timepoints, use of ultrasonography to measure the IRD, and a blinded assessor. Both primiparous and parous women, with both single and multiple pregnancies, were included in this study. This may have had a positive effect on the representativeness of the general pregnant population. However, all participants were self-sampled, more than half reported to have higher education and ethnicity was not reported which may have reduced the representativeness. This study did not differentiate between sex and gender identification, as all participants were recruited using the concept ‘pregnant women’ and recorded as such. This may limit the scope of the findings to this specific population. Moreover, individuals with pregnancy related serious illnesses regarding both fetus and/or mother, or chronic physical or mental illness were excluded from participation. This raises ethical concerns as it may contribute to marginalization of vulnerable groups and limit their access to health services. However, it was considered necessary to maintain these exclusion criteria as these individuals may have required specialized care that this project could not provide for (ethically and/or financially), and/or their participation may have introduced safety risks for both them and others.

As some of the questionnaires and measures used in this study have not been validated for a pregnant population, the interpretation of the results ought to reflect this. To our knowledge there is a lack of standardized and validated tools specific to a pregnant population with low back pain and hypermobility. Careful considerations were taken when these measures were included. Both the Modified Oswestry Disability Index and the 5PQ have been validated on similar populations (women of reproductive age), and as data was collected during the early stages of 2. trimester the impact of pregnancy was not too profound, it was assumed that these tools would provide useful data, though with limitations [25, 26].

The small sample size may pose a limitation to the study, so insignificant associations should be interpreted with caution. Only two participants carried more than one fetus, and a multiple pregnancy may influence the IRD differently compared to a singleton, and to avoid potential confounding factors this data was not included in the analysis. Further studies should be carried out on a significantly larger population of multiple pregnancies to increase the precision of the estimates. Moreover, some of the subgroups were not large enough to provide meaningful results in the linear regression analyses, but should, however, be examined further in larger studies. All variables except for IRD, waist circumference and symphysis fundus height were self-reported, which may be a limitation due to response bias. A final limitation is the lack of pre-pregnancy IRD measurements, as the relation between three measurement points of the IRD provided further knowledge of the development of IRD during pregnancy [4].

## Conclusion

IRD early in pregnancy was strongly associated with IRD at gestation week 37 in a statistical model including BMI, waist circumference, parity, stretch marks and varicose veins. Interestingly, factors that are often presented as related to IRD, such as height and abdominal exercise were not associated with IRD late in pregnancy. As this was a novel and exploratory study with significant limitations, clinical implications are limited except that for each 1 mm increase in baseline IRD, there was an average increase of 0.8 mm in IRD at gestation week 37. Approximately 38% of the variance in IRD at gestation week 37 is explained by baseline IRD and other included variables. Further, longitudinal studies with larger sample sizes are warranted to investigate predictors of large IRDs among pregnant women.

## Electronic supplementary material

Below is the link to the electronic supplementary material.

Supplementary Material 1

Supplementary Material 2

## Data Availability

The data that support the findings of this study are not openly available due to reasons of sensitivity and are available from the corresponding author upon reasonable request. Data are located in controlled access data storage at University of Bergen, Norway.
