# Supplementary material for: Associations with the inter-recti distance at gestation week 37: a prospective cohort study among healthy pregnant women
Source: BMC Pregnancy Childbirth. 2025 May 29;25:630. doi: 10.1186/s12884-025-07741-7 (PMC12123851; doi:10.1186/s12884-025-07741-7)
Supplement: Supplementary file 1 — Supplementary Material 1 [file 12884_2025_7741_MOESM1_ESM.pdf]

Supplemetary file 1. Baseline Questionnaire

BASELINE QUESTIONNAIRE

Date:

Study:

Participant ID:

Gestation week:

1. Height:

2. Weight (at present):

EDUCATION

3. Level of education:

☐Primary / secondary

☐College

☐University, bachelor

☐University, masters/PhD

PREGNANCY

4. Due date present pregnancy:

5. Number of fetuses present pregnancy:

6. Number of children:

7. Number of births:

8. Date of last birth:

9. Delivery mode last birth:

☐Vaginal

☐C-section

YOUR HEALTH

10. How is your health now:

☐Poor

☐Not good

☐Good

☐Very good

11. Do you smoke:

☐Yes, daily

☐Yes, occasionally

☐No

12. Do you have stretchmarks:

☐No

☐Yes

If yes, where?

13. Do you have varicose veins:

☐No

☐yes

If yes, where?

14. Do you have a rheumatological disease/condition?

☐No

☐Yes

If yes, please name the disease/condition?

15. Hypermobility

15a. Can you now (or could you ever) place your hands flat on the floor without bending your knees?

☐Yes

☐No

15b. Can you now (or could you ever) bend your thumb to touch your forearm?

☐Yes

☐No

15c. As a child did you amuse your friends by contorting your body into strange shapes OR could you do the splits?

☐Yes

☐No

15d. As a child or teenager did your shoulder or kneecap dislocate on more than one occasion?

☐Yes

☐No

15e. Do you consider yourself double-jointed?

☐Yes

☐No

PHYSICAL ACTIVITY/EXERCISE

16. How often have you been physical active in the last for 4 weeks?

☐Never

☐Less than once a week

☐Once a week

☐2-3 times a week

☐Almost every day

17. Have you performed abdominal exercises in the last 4 weeks?

☐Never

☐1-3 times

☐Once a week

☐Twice a week

☐Three or more times a week

18. Have you performed pelvic floor exercises in the last 4 weeks?

☐Never

☐Once a week

☐1-2 times a week

☐3 times a week

☐Daily

19. Lifting. Lifting more than 5 kg is considered a heavy load.

19a. Do you do heavy lifting at work:

☐No

☐Yes

If yes:

☐20 times or less a week

☐More than 20 times a week

19.b. Do you do heavy lifting at home:

☐No

☐Yes

If yes:

☐20 times or less a week

☐More than 20 times a week

URINARY INCONTINENS

20. How often do you leak urine? – Tick one box

☐Never

☐About once a week or less often

☐2 – 3 times a week

☐About one a day

☐Several times a day

☐All the time

21. We would like to know how much urine you think leaks. How much urine do you usually leak (whether you wear protection or not)? Tick one box

☐None

☐A small amount

☐A moderate amount

☐A large amount

22. Overall, how much does leaking urine interfere with your everyday life? Please ring a number between 0 (not at all) and 10 (a great deal)

0	1	2	3	4	5	6	7	8	9	10
Not at all										A great deal

LOW BACK PAIN

This questionnaire has been designed to give us information as to how your back or leg pain is affecting your ability to manage in everyday life. Please answer by checking ONE box in each section for the statement which best applies to you. We realise you may consider that two or more statements in any one section apply but please just shade out the spot that indicates the statement which most clearly describes your problem.

Section 1 – Pain intensity

☐I have no pain at the moment
☐The pain is very mild at the moment
☐The pain is moderate at the moment
☐The pain is fairly severe at the moment
☐The pain is very severe at the moment
☐The pain is the worst imaginable at the moment

Section 2 – Personal care (washing, dressing etc)

☐ I can look after myself normally without causing extra pain
☐I can look after myself normally, but it causes extra pain
☐ It is painful to look after myself and I am slow and careful
☐ I need some help but manage most of my personal care
☐ I need help every day in most aspects of self-care
☐ I do not get dressed, I wash with difficulty and stay in bed

Section 3 – Lifting

☐I can lift heavy weights without extra pain
☐I can lift heavy weights, but it gives extra pain
☐Pain prevents me from lifting heavy weights off the floor, but I can manage if they are conveniently placed eg. on a table
☐Pain prevents me from lifting heavy weights, but I can manage light to medium weights if they are conveniently positioned
☐I can lift very light weights
☐I cannot lift or carry anything at all

Section 4 – Walking*

☐Pain does not prevent me walking any distance
☐Pain prevents me from walking more than 2 kilometres
☐Pain prevents me from walking more than 1 kilometre
☐Pain prevents me from walking more than 500 metres
☐I can only walk using a stick or crutches
☐I am in bed most of the time

Section 5 – Sitting

☐I can sit in any chair as long as I like
☐I can only sit in my favourite chair as long as I like
☐Pain prevents me sitting more than one hour
☐Pain prevents me from sitting more than 30 minutes
☐Pain prevents me from sitting more than 10 minutes
☐Pain prevents me from sitting at all

Section 6 – Standing

☐I can stand as long as I want without extra pain
☐I can stand as long as I want but it gives me extra pain
☐Pain prevents me from standing for more than 1 hour
☐Pain prevents me from standing for more than 3 minutes
☐Pain prevents me from standing for more than 10 minutes
☐Pain prevents me from standing at all

Section 7 – Sleeping

☐My sleep is never disturbed by pain
☐My sleep is occasionally disturbed by pain
☐Because of pain I have less than 6 hours sleep
☐Because of pain I have less than 4 hours sleep
☐Because of pain I have less than 2 hours sleep
☐Pain prevents me from sleeping at all

Section 8 – Sex life (if applicable)

☐My sex life is normal and causes no extra pain
☐My sex life is normal but causes some extra pain
☐My sex life is nearly normal but is very painful
☐My sex life is severely restricted by pain
☐My sex life is nearly absent because of pain
☐Pain prevents any sex life at all

Section 9 – Social life

☐My social life is normal and gives me no extra pain
☐My social life is normal but increases the degree of pain
☐Pain has no significant effect on my social life apart from limiting my more energetic interests eg, sport
☐Pain has restricted my social life, and I do not go out as often
☐Pain has restricted my social life to my home
☐I have no social life because of pain

Section 10 – Travelling

☐I can travel anywhere without pain
☐I can travel anywhere but it gives me extra pain
☐Pain is bad but I manage journeys over two hours
☐Pain restricts me to journeys of less than one hour
☐Pain restricts me to short necessary journeys under 30 minutes
☐Pain prevents me from travelling except to receive treatment

PELVIC GIRDLE PAIN

To what extent do you find it problematic to carry out the activities listed below because of pelvic girdle pain? For each activity tick the box that best describes how you are today.

How problematic is it for you because of your pelvic girdle pain to:	Not at all (0)	To a small extent (1)	To some extent (2)	To a large extent (3)
Dress yourself	☐	☐	☐	☐
Stand for less than 10 minutes	☐	☐	☐	☐
Stand for more than 60 minutes	☐	☐	☐	☐
Bend down	☐	☐	☐	☐
Sit for less than 10 minutes	☐	☐	☐	☐
Sit for more than 60 minutes	☐	☐	☐	☐
Walk for less than 10 minutes	☐	☐	☐	☐
Walk for more than 60 minutes	☐	☐	☐	☐
Climb stairs	☐	☐	☐	☐
Do housework	☐	☐	☐	☐
Carry light objects	☐	☐	☐	☐
Carry heavy objects	☐	☐	☐	☐
Get up/sit down	☐	☐	☐	☐
Push a shopping cart	☐	☐	☐	☐
Run	☐	☐	☐	☐
Carry out sporting activities*	☐	☐	☐	☐
Lie down	☐	☐	☐	☐
Roll over in bed	☐	☐	☐	☐
Have a normal sex life*	☐	☐	☐	☐
Push something with one foot	☐	☐	☐	☐
*If not applicable, mark box to the right.				
How much pain do you experience:	**None (0)**	**Some (1)**	**Moderate (2)**	**Considerable (3)**
In the morning	☐	☐	☐	☐
In the evening	☐	☐	☐	☐
To what extent because of pelvic girdle pain:	**Not at all (0)**	**To a small extent (1)**	**To some extent (2)**	**To a large extent (3)**
Has your leg/have your legs given way?	☐	☐	☐	☐
Do you do things more slowly?	☐	☐	☐	☐
Is your sleep interrupted?	☐	☐	☐	☐

Scoring procedure: the scores were summarized and recalculated to percentage scores from 0 (no problem at all) to 100 (to a large extent).
