# Supplementary figures and images for: Associations with the inter-recti distance at gestation week 37: a prospective cohort study among healthy pregnant women
Source: BMC Pregnancy Childbirth. 2025 May 29;25:630. doi: 10.1186/s12884-025-07741-7 (PMC12123851; doi:10.1186/s12884-025-07741-7)

Association between IRD at baseline and gestation week 37

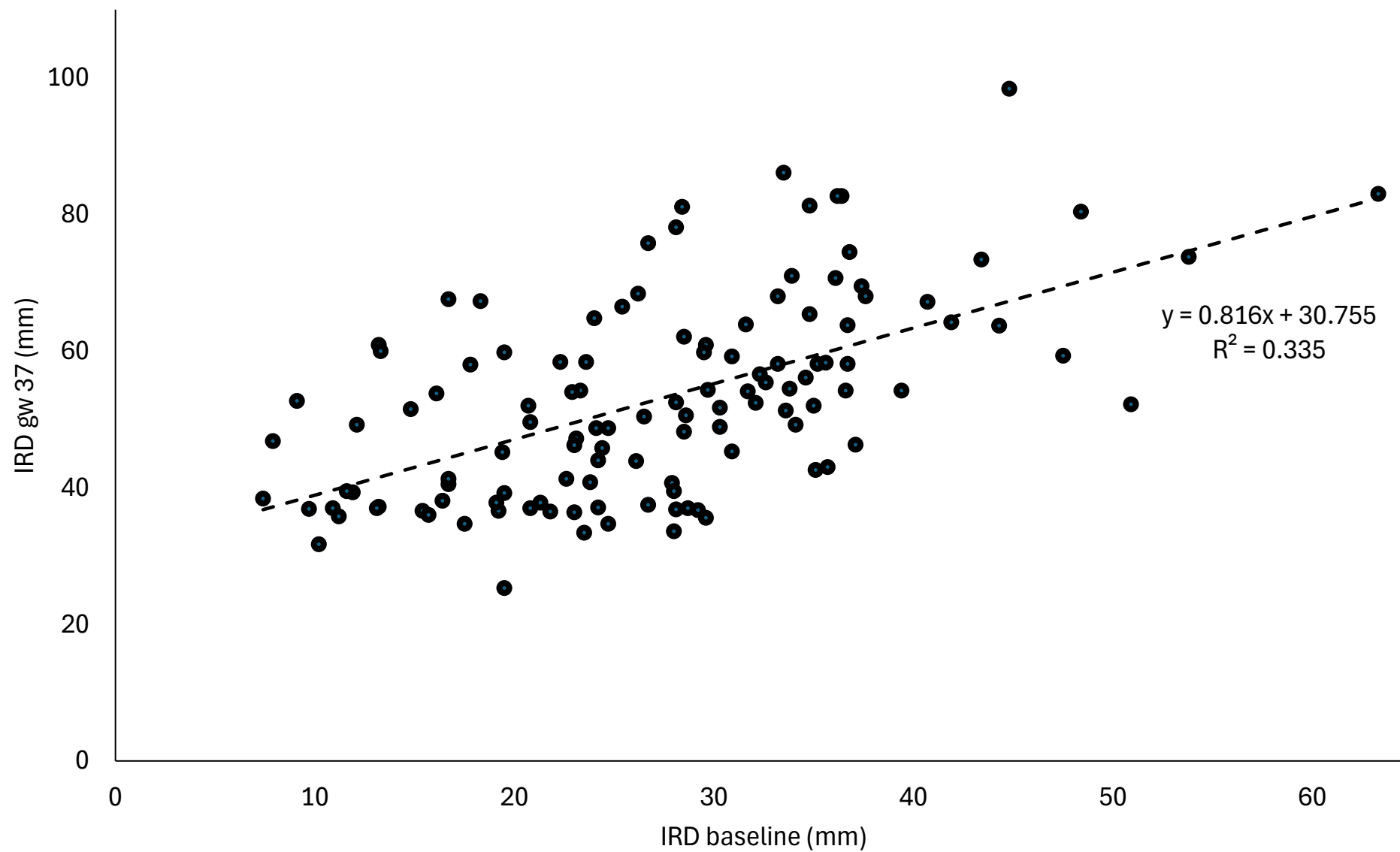

Supplement: Supplementary file 2 — Supplementary Material 2 [file 12884_2025_7741_MOESM2_ESM.pdf]
